# Supplementary material for: Weight perceptions in older adults: findings from the English Longitudinal Study of Ageing
Source: BMJ Open. 2020 Feb 9;10(2):e033773. doi: 10.1136/bmjopen-2019-033773 (PMC7044993; doi:10.1136/bmjopen-2019-033773)
Supplement: Author's manuscript [file bmjopen-2019-033773.draft_revisions.pdf]

BMJ Open

BMJ Open is committed to open peer review. As part of this commitment we make the peer review history of every article we publish publicly available.

When an article is published we post the peer reviewers' comments and the authors' responses online. We also post the versions of the paper that were used during peer review. These are the versions that the peer review comments apply to.

The versions of the paper that follow are the versions that were submitted during the peer review process. They are not the versions of record or the final published versions. They should not be cited or distributed as the published version of this manuscript.

BMJ Open is an open access journal and the full, final, typeset and author-corrected version of record of the manuscript is available on our site with no access controls, subscription charges or pay-per-view fees (http://bmjopen.bmj.com).

If you have any questions on BMJ Open's open peer review process please email info.bmjopen@bmj.com

BMJ Open

Time to reconsider body mass index for defining weight status in old age? Insights from older adults' own perceptions of weight status

Journal:	*BMJ Open*
Manuscript ID	bmjopen-2019-033773
Article Type:	Original research
Date Submitted by the Author:	26-Aug-2019

Complete List of Authors:	Jackson, Sarah ; University College London, Behavioural Science and Health<br>Smith, Lee; Anglia Ruskin University - Cambridge Campus, Department of Life Sciences<br>Steptoe, Andrew; University College London, Epidemiology and Public Health

Keywords:	GERIATRIC MEDICINE, PUBLIC HEALTH, EPIDEMIOLOGY

SCHOLARONE™
Manuscripts

I, the Submitting Author has the right to grant and does grant on behalf of all authors of the Work (as defined in the below author licence), an exclusive licence and/or a non-exclusive licence for contributions from authors who are: i) UK Crown employees; ii) where BMJ has agreed a CC-BY licence shall apply, and/or iii) in accordance with the terms applicable for US Federal Government officers or employees acting as part of their official duties; on a worldwide, perpetual, irrevocable, royalty-free basis to BMJ Publishing Group Ltd ("BMJ") its licensees and where the relevant Journal is co-owned by BMJ to the co-owners of the Journal, to publish the Work in this journal and any other BMJ products and to exploit all rights, as set out in our licence.

The Submitting Author accepts and understands that any supply made under these terms is made by BMJ to the Submitting Author unless you are acting as an employee on behalf of your employer or a postgraduate student of an affiliated institution which is paying any applicable article publishing charge ("APC") for Open Access articles. Where the Submitting Author wishes to make the Work available on an Open Access basis (and intends to pay the relevant APC), the terms of reuse of such Open Access shall be governed by a Creative Commons licence – details of these licences and which Creative Commons licence will apply to this Work are set out in our licence referred to above.

Other than as permitted in any relevant BMJ Author's Self Archiving Policies, I confirm this Work has not been accepted for publication elsewhere, is not being considered for publication elsewhere and does not duplicate material already published. I confirm all authors consent to publication of this Work and authorise the granting of this licence.

For peer review only

Time to reconsider body mass index for defining weight status in old age? Insights from older adults' own perceptions of weight status

Sarah E. Jackson[1], Lee Smith[2], Andrew Steptoe[1]

[1]Department of Behavioural Science and Health, University College London, London, UK

[2] Cambridge Centre for Sport and Exercise Sciences, Anglia Ruskin University, Cambridge, UK

Corresponding author: Sarah E Jackson, PhD. Department of Behavioural Science and Health, University College London, 1-19 Torrington Place, London WC1E 6BT

Tel: (44) 207 679 3179

Fax: (44) 207 916 8354

s.e.jackson@ucl.ac.uk

Word count: 3,465

For peer review only

For peer review only - http://bmjopen.bmj.com/site/about/guidelines.xhtml

Abstract

Objectives: Questions have been raised about the appropriateness of conventional body mass index (BMI) cut-offs for defining weight status in later life given evidence of lack of sensitivity in classifying overweight and undernutrition. This study aimed to explore weight perceptions in a large, nationally-representative sample of older adults, and the extent to which they differ according to age and perceived health status.

Setting: England.

Participants: 5,240 older adults (≥50y) participating in the English Longitudinal Study of Ageing (2016/17).

Main outcome measures: Weight perception was self-reported as too heavy, too light, or about right.

Results: The majority of older adults endorsed a weight perception that matched their (objectively measured) BMI classification. However, one in ten (9.9%) older adults classified by BMI as normal-weight (18.5-24.9kg/m^2) felt too light, with women at the upper end of the older age spectrum (OR=1.04, 95%CI=1.01-1.09), and men (OR=3.70, 95%CI=1.88-7.28) and women (OR=2.61, 95%CI=1.27-5.35) in poorer health more likely to do so. Almost half (44.8%) of older adults classified as overweight (25-29.9kg/m^2) and one in ten (10.3%) classified as obese (≥30kg/m^2) felt about the right weight, with this observed more frequently among men and women at the upper end of the older age spectrum (OR range 1.04-1.06).

Conclusion: Older adults' perceptions of their own weight generally correspond with traditional BMI cut-offs for normal-weight, overweight, and obesity. However, a substantial minority 'underestimate' their weight status, with those at the upper end of the age spectrum and those in poorer health more likely to do so. These findings add further weight to questions raised about the appropriateness of standard definitions of overweight and obesity for older adults and calls to consider development of age-specific recommendations for healthy weight.

Key words: body mass index; weight perceptions; population-based study; obesity paradox

For peer review only - http://bmjopen.bmj.com/site/about/guidelines.xhtml

Strengths and limitations of the study

Data were from a large, representative sample of older adults in England.

Height and weight were objectively measured for calculation of body mass index.

However, there was a substantial amount of missing data, so findings may not generalise to the entire population.

If those who were more concerned about their weight were more likely to decline to be measured, our results may underestimate the proportion of older adults across all weight groups who consider themselves to be too heavy.

For peer review only - http://bmjopen.bmj.com/site/about/guidelines.xhtml

Introduction

The global obesity epidemic and ageing population are major public health concerns. With excess weight becoming increasingly 'normal', public perceptions of what constitutes a healthy body weight have become more inaccurate over time, with increasing numbers perceiving a body mass index (BMI) in the overweight or obese range (≥25 kg/m^2) to be 'about right' [1]. The numbers of older adults with overweight (BMI 25-29.9 kg/m^2) and obesity (BMI ≥30 kg/m^2) are rising rapidly, due to concurrent increases in the number of adults who reach older age and the proportion who carry excess weight [2–4]. However, there is controversy over the appropriateness of conventional BMI cut-offs for defining weight status in later life [5–9] because of their low sensitivity in identifying older adults at risk of undernutrition [5] or obesity [6,8], and paradoxical evidence of increased survival [9] and better functional status [10] among older people with an overweight compared with normal-weight (18.5-24.9 kg/m^2) BMI. Understanding how older adults perceive their own weight status, and the extent to which this is influenced by their age and health status, is important for gaining insight into this so-called age-associated 'obesity paradox' [9,11,12] from a patient perspective and informing targeted recommendations and interventions to promote healthy weight in later life.

Older adults' weight perceptions may be influenced by several social and physiological factors. On a societal level, there are strong preferences for slimness in women and lean muscularity in men [13]. Some evidence suggests that older people may be less influenced by, and feel less pressure to attain, cultural body weight ideals [14,15]. Nonetheless, body dissatisfaction is evident in mid- and later life [16–20] and may even increase as age-related changes in body composition widen the discrepancy between ideal and actual body image [21]. Ageing is associated with an increase in fat mass, loss of lean muscle mass, and redistribution of adipose tissue to the abdominal region [22]; changes that may occur without concomitant changes in body weight and BMI [23].

While few previous studies have examined older adults' weight perceptions, those that have indicate age-related changes in perceptions of body weight in relation to actual BMI category. For example, in a sample of adult Korean women (*n*=8,906, age 20-79 years), older women were more likely than younger women to underestimate their weight status relative to their actual BMI category (50.7% of 70-79 year olds vs. 12.6% of 20-29 year olds) and less likely to overestimate their weight status (7.4% vs. 18.7%, respectively) [24]. Similarly, in a study of older Dutch men and women (*n*=1,295, age 60-96 years), the proportion of women who underestimated their weight increased with age (OR=2.97, 95% CI 1.59-5.57 for 80-96 year olds vs. 60-69 year olds), although no such pattern was found for men [25]. However, it was not clear from these studies

For peer review only - http://bmjopen.bmj.com/site/about/guidelines.xhtml

whether these differences are driven by differences in health status between older and younger people, as might be expected by evidence related to the obesity paradox. If underestimation of body weight is more pronounced for those who perceive their health to be poor, it would suggest that the optimal BMI for older people may be higher than for younger adults and further call into question the suitability of existing BMI cut-offs to define weight status in later life.

The present study therefore aimed to explore perceptions of weight in a large, nationally-representative sample of older adults living in England, and the extent to which they differ according to age and perceived health status.

For peer review only

Method

Study population

Data were from the English Longitudinal Study of Ageing (ELSA), a panel study of men and women aged ≥50 years living in England. Full details of the study's sampling procedure and methods are available elsewhere [26]. The present analyses use data collected in the eighth wave of ELSA (collected 2016/17), as this is the only wave in which weight perceptions have been assessed. Of the 8,445 participants interviewed, 5,240 (62.0%) had complete data on all variables of interest and comprised our analytic sample.

Measures

Weight perception

Weight perception was assessed with the question "*Given your age and height, would you say that you are about the right weight, too heavy, or too light?*".

Anthropometric data

Weight was measured by nurses to the nearest 0.1 kg using portable electronic scales. Height was measured in Wave 6 (it was not included in the Wave 8 assessment) to the nearest millimetre using a portable stadiometer. Nurses recorded any factors that might have compromised the reliability of the measurements (e.g. participant was stooped/unwilling to remove shoes) and these cases were excluded. BMI was calculated as weight in kilograms divided by the square of height in metres, and categorised as underweight

For peer review only - http://bmjopen.bmj.com/site/about/guidelines.xhtml

(<18.5 kg/m^2), normal-weight (18.5 to <25 kg/m^2), overweight (25 to <30 kg/m^2) and obese (≥30 kg/m^2). We excluded participants with a BMI in the underweight range because there were insufficient numbers for meaningful analysis as a separate group (*n*=64, 1.2% of otherwise eligible sample).

For some descriptive analyses, normal-weight participants were divided into those with a BMI in the lower half of the normal-weight range (BMI 18.5 to <21.75 kg/m^2; 'lower normal-weight') and those in the upper half (BMI 21.75 to <30 kg/m^2; 'upper normal-weight').

Sociodemographic information

Information on age, sex, ethnicity (white vs. non-white), and SES was recorded. SES was indexed using the short version of the NSSEC 3-category classification of the present or most recent occupation and categorised as higher (managerial/professional occupations), intermediate (intermediate occupations), and lower (routine/manual occupations) [27].

Perceived health and comorbidities

Self-rated health was assessed using a single item: *"Would you say your health is… poor/fair/good/very good/excellent?"* We analysed the proportion of individuals rating their health as fair/poor, as is commonly done in analyses of this variable [28–30].

Information about five doctor-diagnosed chronic diseases that may cause weight loss (cancer, stroke, chronic lung disease, diabetes, and arthritis) was self-reported and the number of reported conditions were summed to create a chronic health condition index ranging from 0-5. Because scores were highly skewed, we dichotomised this variable to distinguish between 0 and ≥1 health conditions.

Statistical analysis

Analyses were performed using IBM SPSS Statistics 24. In order to produce representative estimates for older adults in the English population, data were weighted to correct for sampling probabilities and to match the English population on age and sex. The weights accounted for the differential probability of being included in Wave 8 of ELSA.

Analyses were performed separately for men and women. We tested sex differences in sociodemographic, anthropometric, self-rated health, and weight perception variables using *t*-tests for continuous variables and Pearson's chi-square analyses for categorical variables. We used multivariable logistic regression to

For peer review only - http://bmjopen.bmj.com/site/about/guidelines.xhtml

identify independent associations with perception of weight as (i) 'too heavy' or (ii) 'too light' among normal-weight participants, and associations with perception of weight as 'about right' among overweight and obese participants. Variables included in the models were age, ethnicity, SES, self-rated health, and BMI. In order to evaluate whether perception of weight as too light among normal-weight participants was associated with health conditions that may cause weight loss, we also adjusted for chronic health conditions in this model. Results are reported as odds ratios (ORs) with 95% confidence intervals (CIs).

Patient and public involvement

Patients and the public were not involved in this research.

Results

There were 2,363 men and 2,911 women in the sample. Descriptive characteristics overall and by sex are shown in Table 1. The mean age was 70.0 (SD 7.8) years. The majority of participants were white (96.2%) and rated their health as good, very good, or excellent (72.7%) despite many having one or more chronic health conditions (58.0%). There was fairly even distribution across socioeconomic groups (32.4% higher SES, 26.8% intermediate SES, 40.8% lower SES), although women were less likely than men to be in the higher SES group (26.6% vs. 38.8%, $p<0.001$). Just over a quarter (27.5%) of participants had a BMI in the normal-weight range, 40.3% were overweight, and a further 32.3% had obesity. On average, men were significantly taller and heavier than women ($p<0.001$). While there was no significant sex difference in mean BMI ($p=0.116$), men were more likely than women to have a BMI placing them in the overweight range (46.1% vs. 35.0%) while women were more likely than men to have a normal-weight (30.8% vs. 23.8%) or obese BMI (34.2% vs. 30.1%, $p<0.001$).

Weight perception among normal-weight older adults

Figure 1 summarises the distribution of weight perceptions by sex and weight status. In the normal-weight category, the majority (80.3%) of participants thought they were about the right weight, but 9.9% thought they were too light, and 9.7% thought they were too heavy. Normal-weight women were significantly more likely than normal-weight men to consider themselves to be too heavy (12.6% vs. 5.6%, $p<0.001$) and less likely to consider themselves too light (7.3% vs. 13.7%, $p<0.001$).

For peer review only - http://bmjopen.bmj.com/site/about/guidelines.xhtml

For peer review only

Multivariable models testing independent associations of age, sex, ethnicity, SES, and BMI with perception of body weight as too heavy in normal-weight men and women are presented in Table 2 (left panel). There was a significant independent association between BMI and perception of weight as too heavy in both sexes, with each unit increase in BMI associated with 1.76 times higher odds (95% CI 1.18-2.61) of perception of weight as too heavy in men and 2.14 times higher odds (95% CI 1.69-2.72) in women. Some 6.7% of men and 16.8% of women in the upper normal-weight group felt too heavy, compared with just 1.1% of men and 2.5% of women in the lower normal-weight group. Older age was significantly associated with reduced odds of perception of weight as too heavy in women (OR=0.97, 95% CI 0.94-1.00) but not in men. There was no significant independent association between perception of weight as too heavy and ethnicity, SES, or self-rated health in either sex.

Factors independently associated with perception of body weight as too light in normal-weight men and women are shown in Table 2 (right panel). There were strong independent associations with BMI and self-rated health. In both sexes, odds of feeling too light were significantly lower among those with a higher BMI, with each unit increase in BMI associated with a 47% reduction in odds in men (OR=0.53, 95% CI 0.43-0.64) and a 53% reduction in odds in women (OR=0.47, 95% CI 0.38-0.58). Just 6.5% of men and 2.6% of women in the upper normal-weight group felt too light, compared with 43.3% of men and 17.8% of women in the lower normal-weight group. Odds of perception of weight as too light were significantly increased among participants with fair/poor self-rated health (men: OR=3.70, 95% CI 1.88-7.28; women: OR=2.61, 95% CI 1.27-5.35). The presence of one or more chronic health conditions was also independently associated with increased odds of perception of weight as too light in women (OR=2.21, 95% CI 1.03-4.77) but not in men. Older age was associated with increased odds of perception of weight as too light in normal-weight women (OR=1.04, 95% CI 1.01-1.09) but not in men. Non-white ethnicity was associated with increased odds of perception of weight as too light in normal-weight men (OR=6.26, 95% CI 2.09-18.76) but not in women. There was no significant independent association with SES in either sex.

Weight perception among overweight and obese older adults

Among participants with a BMI in the overweight range, 54.3% thought they were too heavy, but 44.8% thought they were about the right weight and 0.9% thought they were too light. Among participants with an obese BMI, 89.4% thought they were too heavy, 10.3% thought they were about the right weight and 0.4% thought they were too light. Women with overweight were more likely than men with overweight to recognise that they were too heavy (63.4% vs. 46.7%, $p<0.001$) and less likely to perceive themselves to be about the right weight (35.7% vs. 52.5%, $p<.001$). Likewise, women with obesity were more likely than men

For peer review only - http://bmjopen.bmj.com/site/about/guidelines.xhtml

with obesity to perceive their weight as too heavy (91.3% vs. 87.0%, *p*=0.012) and less likely to perceive their weight as about right (8.5% vs. 12.4%, *p*=0.020) (Figure 1).

Factors independently associated with perception of body weight as about right in men and women with overweight are summarised in Table 3. Among overweight men and women, there was a strong independent association with BMI: each unit increase in BMI was associated with 48% lower odds of perception of weight as about right in men (OR=0.52, 95% CI 0.47-0.59) and 45% lower odds in women (OR=0.55, 95% CI 0.49-0.63). Older age was also independently associated with increased odds of perception of weight as about right in both sexes (men: OR=1.04, 95% CI 1.02-1.05; women: OR=1.06, 95% CI 1.04-1.09). Non-white ethnicity was associated with increased odds of perception of weight as about right in women (OR=3.80, 95% CI 1.48-9.98) but not in men. An association with SES was also observed in women, with overweight women in the intermediate and lower SES groups significantly more likely to perceive their weight to be about right than those from the higher SES group (intermediate: OR=1.79, 95% CI 1.15-2.80; lower: OR=1.73, 95% CI 1.12-2.66). There was no significant association with SES in men. There was no significant independent association with self-rated health in either sex.

Factors independently associated with perception of body weight as about right in men and women with obesity are shown in Table 4. Older age was significantly associated with increased odds of perception of body weight as about right in both men (OR=1.04, 95% CI 1.01-1.08) and women (OR=1.04, 95% CI 1.01-1.08). Higher BMI was associated with 24% lower odds of perception of body weight as about right in men (OR=0.76, 95% CI 0.67-0.86) but no significant association with BMI was observed in women. Non-white ethnicity was significantly associated with increased odds of perception of body weight as about right in women (OR=2.72, 95% CI 1.02-7.27) but not in men. No significant association between perception of body weight as about right and either SES or self-rated health was observed in either sex.

Discussion

In a large, representative sample of older adults living in England, we found that weight perceptions broadly corresponded to participants' actual weight status as defined by widely used BMI cut-offs: 80% of older adults with a BMI in the normal-weight range thought they were about the right weight, and over 50% of older adults with a BMI in the overweight range and almost 90% of those with a BMI in the obese range thought they were too heavy. However, a substantial number of older adults either under- or overestimated

For peer review only - http://bmjopen.bmj.com/site/about/guidelines.xhtml

their weight status relative to their BMI category. One in ten participants with a normal-weight BMI thought they were too heavy and one in ten thought they were too light. Almost half of participants with an overweight BMI thought they were about the right weight, as did one in ten of those with an obese BMI.

As has been observed in previous research examining weight perceptions in younger samples [1,31,32], there were systematic differences between men and women's weight perceptions. Across all BMI categories, men were consistently more likely than women to underestimate their weight status, and normal-weight women were more likely than normal-weight men to report feeling too heavy. There were also some differences in weight perceptions by ethnicity and SES, with people from non-white ethnic groups and intermediate and lower SES groups more likely to underestimate their weight status, as has been shown previously in younger samples [31,32], although these differences were not consistently observed across BMI categories or sexes.

Importantly, there were also clear age-related differences in weight perceptions across all BMI categories. In men and women with an overweight or obese BMI, the odds of feeling about the right weight increased with advancing age. In women with a normal-weight BMI, the odds of feeling too light increased and the odds of feeling too heavy decreased with age, although there were no significant differences by age in men. These findings are suggestive of a higher ideal weight at older ages. This is consistent with previous studies that have shown people, particularly women, tend to endorse a slightly larger and more curvaceous body shape as they get older [16,33]. It is possible that older people are aware that having an overweight or obese BMI is not necessarily a bad thing at older ages. Alternatively, as people get older they may give up the effort to reduce weight at higher BMIs, perhaps because other health issues become more salient for them, or because they are less interested in self-presentation and striving for a slimmer physique [20].

There was also a significant association between self-rated health and odds of feeling too light among men and women with a normal-weight BMI. Those who rated their health as fair or poor had around three times higher odds of feeling too light at a normal-weight BMI than those who rated their health as good, very good, or excellent. In addition, women with a normal-weight BMI who had at least one chronic condition were also more likely to consider themselves to be too light, although there was no difference in men. These results could be interpreted as suggesting that the ideal weight may be higher for older adults in poorer health than for those in good health. There is a vast literature documenting an obesity paradox in chronic conditions including cardiovascular disease [34], cancer [35], kidney disease [36], and lung disease [37], whereby patients who carry excess weight have better outcomes than those with a normal-weight BMI. It has also been suggested that stress related to negative body image perception may have a causal role in the

For peer review only - http://bmjopen.bmj.com/site/about/guidelines.xhtml

development of poor health [38]. However, weight perceptions did not differ significantly by self-rated health among participants with a BMI in the overweight or obese range, indicating that older people with poor health were no more likely to feel they were about the right weight at an overweight or obese BMI than those in good health, so any influence of health status on ideal weight appears to be restricted to the lower end of the weight spectrum. An alternative explanation is that the association between poorer self-rated health and increased likelihood of feeling too light might secondary to illness. Older people with serious chronic illnesses often lose weight, so the fact they think they are too light might be a reflection of this concern.

Taken together, these findings are consistent with previous literature that has questioned the use of conventional BMI definitions of weight status in older populations [5–9]. If standard BMI cut-offs do not accurately predict health risks or survival at older ages, and older people are increasingly likely as they get older to consider themselves too light at a ‘normal-weight’ BMI and ‘about right’ at an overweight BMI, there is a need to consider to how useful these thresholds are for categorising weight status in this age group. A mismatch between the standard BMI cut-offs and the optimal weight range for health also has implications for recommendations on healthy weight. Currently, guidance issued by official bodies such as the UK National Health Service [39] and US Centers for Disease Control and Prevention [40] does not differ according to age group or health status. However, many health professionals are reluctant to recommend weight loss for older patients with an overweight BMI [41,42] and there have been calls to reconsider the standards for ideal weight at older ages and develop age-specific recommendations [43–45]. The present results provide further support for this, demonstrating clear changes in older adults’ perceptions of their weight as they get older, with an increasing sense of feeling too light at a normal-weight BMI and about the right weight at an overweight BMI with advancing age.

Strengths of the present study include a large, representative sample of older adults in England and objective measurements of height and weight. However, findings should be considered in the light of several limitations. There was a substantial amount of missing data, so findings may not generalise to the entire population. Of note, weight measurements were not available for all Wave 8 participants (8% missing). If those who were more concerned about their weight were more likely to decline to be measured, our results may underestimate the proportion of older adults across all weight groups who consider themselves to be too heavy.

For peer review only - http://bmjopen.bmj.com/site/about/guidelines.xhtml

For peer review only

In conclusion, the present results indicate that older adults' perceptions of their own weight generally correspond with traditional BMI cut-offs for normal-weight, overweight, and obesity. However, a substantial minority 'underestimate' their weight status, with those at the upper end of the age spectrum and those in poorer health more likely to do so. These findings add further weight to questions raised about the appropriateness of standard definitions of overweight and obesity for older adults and calls to consider development of age-specific recommendations for healthy weight.

For peer review only

For peer review only - http://bmjopen.bmj.com/site/about/guidelines.xhtml

References

1. Johnson F, Cooke L, Croker H, Wardle J. Changing perceptions of weight in Great Britain: comparison of two population surveys. *BMJ*. 2008;337(jul10 1):a494-a494. doi:10.1136/bmj.a494

2. Vincent GK, Velkoff VA. *The next Four Decades: The Older Population in the United States: 2010 to 2050*. US Department of Commerce, Economics and Statistics Administration, US Census Bureau; 2010.

3. Wang YC, McPherson K, Marsh T, Gortmaker SL, Brown M. Health and economic burden of the projected obesity trends in the USA and the UK. *The Lancet*. 2011;378(9793):815-825. doi:10.1016/S0140-6736(11)60814-3

4. Villareal DT, Apovian CM, Kushner RF, Klein S. Obesity in Older Adults: Technical Review and Position Statement of the American Society for Nutrition and NAASO, The Obesity Society. *Obes Res*. 2005;13(11):1849-1863. doi:10.1038/oby.2005.228

5. Beck AM, Ovesen L. At which body mass index and degree of weight loss should hospitalized elderly patients be considered at nutritional risk? *Clin Nutr Edinb Scotl*. 1998;17(5):195-198.

6. Sardinha LB, Teixeira PJ. Obesity screening in older women with the body mass index: A receiver operating characteristic (ROC) analysis. *Sci Sports*. 2000;15(4):212-219. doi:10.1016/S0765-1597(00)80008-8

7. Cook Z, Kirk S, Lawrenson S, Sandford S. Use of BMI in the assessment of undernutrition in older subjects: reflecting on practice. *Proc Nutr Soc*. 2005;64(3):313-317. doi:10.1079/PNS2005437

8. Vasconcelos F de AG de, Cordeiro BA, Rech CR, Petroski EL. Sensitivity and specificity of the body mass index for the diagnosis of overweight/obesity in elderly. *Cad Saúde Pública*. 2010;26:1519-1527. doi:10.1590/S0102-311X2010000800006

9. Oreopoulos A, Kalantar-Zadeh K, Sharma AM, Fonarow GC. The Obesity Paradox in the Elderly: Potential Mechanisms and Clinical Implications. *Clin Geriatr Med*. 2009;25(4):643-659. doi:10.1016/j.cger.2009.07.005

10. Bahat G, Tufan F, Saka B, et al. Which body mass index (BMI) is better in the elderly for functional status? *Arch Gerontol Geriatr*. 2012;54(1):78-81. doi:10.1016/j.archger.2011.04.019

11. Wang S, Ren J. Obesity Paradox in Aging: From Prevalence to Pathophysiology. *Prog Cardiovasc Dis*. 2018;61(2):182-189. doi:10.1016/j.pcad.2018.07.011

12. Chapman IM. Obesity paradox during aging. *Interdiscip Top Gerontol*. 2010;37:20-36. doi:10.1159/000319992

13. Swami V. Cultural Influences on Body Size Ideals. *Eur Psychol*. 2015;20(1):44-51. doi:10.1027/1016-9040/a000150

For peer review only - http://bmjopen.bmj.com/site/about/guidelines.xhtml

14. Bedford JL, Johnson CS. Societal Influences on Body Image Dissatisfaction in Younger and Older Women. *J Women Aging*. 2006;18(1):41-55. doi:10.1300/J074v18n01_04

15. Dunkel TM, Davidson D, Qurashi S. Body satisfaction and pressure to be thin in younger and older Muslim and non-Muslim women: The role of Western and non-Western dress preferences. *Body Image*. 2010;7(1):56-65. doi:10.1016/j.bodyim.2009.10.003

16. Tiggemann M. Body image across the adult life span: stability and change. *Body Image*. 2004;1(1):29-41. doi:10.1016/S1740-1445(03)00002-0

17. Webster J, Tiggemann M. The Relationship Between Women's Body Satisfaction and Self-Image Across the Life Span: The Role of Cognitive Control. *J Genet Psychol*. 2003;164(2):241-252. doi:10.1080/00221320309597980

18. Lamb CS, Jackson LA, Cassiday PB, Priest DJ. Body figure preferences of men and women: A comparison of two generations. *Sex Roles*. 1993;28(5-6):345-358. doi:10.1007/BF00289890

19. Rozin P, Fallon A. Body image, attitudes to weight, and misperceptions of figure preferences of the opposite sex: a comparison of men and women in two generations. *J Abnorm Psychol*. 1988;97(3):342-345.

20. Jackson SE, Holter L, Beeken RJ. 'Just because I'm old it doesn't mean I have to be fat': a qualitative study exploring older adults' views and experiences of weight management. *BMJ Open*. 2019;9(2):e025680. doi:10.1136/bmjopen-2018-025680

21. Tiggemann M. Body-size dissatisfaction: Individual differences in age and gender, and relationship with self-esteem. *Personal Individ Differ*. 1992;13(1):39-43. doi:10.1016/0191-8869(92)90215-B

22. St-Onge M-P, Gallagher D. Body composition changes with aging: The cause or the result of alterations in metabolic rate and macronutrient oxidation? *Nutr Burbank Los Angel Cty Calif*. 2010;26(2):152-155. doi:10.1016/j.nut.2009.07.004

23. St-Onge M-P. Relationship between body composition changes and changes in physical function and metabolic risk factors in aging. *Curr Opin Clin Nutr Metab Care*. 2005;8(5):523-528.

24. Park B, Cho HN, Choi E, et al. Self-perceptions of body weight status according to age-groups among Korean women: A nationwide population-based survey. *PLOS ONE*. 2019;14(1):e0210486. doi:10.1371/journal.pone.0210486

25. Monteagudo Sánchez C, Dijkstra SC, Visser M. Self-perception of body weight status in older Dutch adults. *J Nutr Health Aging*. 2015;19(6):612-618. doi:10.1007/s12603-015-0486-z

26. Steptoe A, Breeze E, Banks J, Nazroo J. Cohort profile: the English Longitudinal Study of Ageing. *Int J Epidemiol*. 2013;42(6):1640-1648. doi:10.1093/ije/dys168

27. Rose D, Pevalin DJ. *A Researcher's Guide to the National Statistics Socio-Economic Classification*. SAGE Publications Ltd; 2003.

For peer review only - http://bmjopen.bmj.com/site/about/guidelines.xhtml

28. Steptoe A, Jackson SE. The Life Skills of Older Americans: Association with Economic, Psychological, Social, and Health Outcomes. *Sci Rep*. 2018;8(1):9669. doi:10.1038/s41598-018-27909-w

29. DeSalvo KB, Bloser N, Reynolds K, He J, Muntner P. Mortality prediction with a single general self-rated health question. A meta-analysis. *J Gen Intern Med*. 2006;21(3):267-275. doi:10.1111/j.1525-1497.2005.00291.x

30. Jackson SE, Hackett RA, Steptoe A. Associations between age discrimination and health and wellbeing: cross-sectional and prospective analysis of the English Longitudinal Study of Ageing. *Lancet Public Health*. 2019;in press.

31. Jackson SE, Johnson F, Croker H, Wardle J. Weight perceptions in a population sample of English adolescents: cause for celebration or concern? *Int J Obes*. 2015;39(10):1488-1493. doi:10.1038/ijo.2015.126

32. Yaemsiri S, Slining MM, Agarwal SK. Perceived weight status, overweight diagnosis, and weight control among US adults: the NHANES 2003–2008 Study. *Int J Obes*. 2011;35(8):1063-1070. doi:10.1038/ijo.2010.229

33. Clarke LCH. Beauty in Later Life: Older Women's Perceptions of Physical Attractiveness. *Can J Aging Rev Can Vieil*. 2002;21(3):429-442. doi:10.1017/S0714980800001744

34. Lavie CJ, Milani RV, Ventura HO. Obesity and the "Obesity Paradox" in Cardiovascular Diseases. *Clin Pharmacol Ther*. 2011;90(1):23-25. doi:10.1038/clpt.2011.87

35. Lennon H, Sperrin M, Badrick E, Renehan AG. The Obesity Paradox in Cancer: a Review. *Curr Oncol Rep*. 2016;18(9):56. doi:10.1007/s11912-016-0539-4

36. Park J, Ahmadi S-F, Streja E, et al. Obesity Paradox in End-Stage Kidney Disease Patients. *Prog Cardiovasc Dis*. 2014;56(4):415-425. doi:10.1016/j.pcad.2013.10.005

37. Chittal P, Babu AS, Lavie CJ. Obesity Paradox: Does Fat Alter Outcomes in Chronic Obstructive Pulmonary Disease? *COPD J Chronic Obstr Pulm Dis*. 2015;12(1):14-18. doi:10.3109/15412555.2014.915934

38. Muennig P, Jia H, Lee R, Lubetkin E. I Think Therefore I Am: Perceived Ideal Weight as a Determinant of Health. *Am J Public Health*. 2008;98(3):501-506. doi:10.2105/AJPH.2007.114769

39. NHS Digital. Healthy weight. nhs.uk. https://www.nhs.uk/live-well/healthy-weight/. Published March 17, 2019. Accessed March 17, 2019.

40. Centers for Disease Control and Prevention. Assessing Your Weight | Healthy Weight. https://www.cdc.gov/healthyweight/assessing/index.html. Published March 6, 2019. Accessed March 17, 2019.

41. Houston DK, Nicklas BJ, Zizza CA. Weighty Concerns: The Growing Prevalence of Obesity among Older Adults. *J Am Diet Assoc*. 2009;109(11):1886-1895. doi:10.1016/j.jada.2009.08.014

For peer review only - http://bmjopen.bmj.com/site/about/guidelines.xhtml

42. Sommers A. U.S. Congressional Research Service: obesity among older Americans. 2011. http://www.legistorm.com/score_crs/show/id/104646.html. Accessed April 18, 2013.

43. Andres R, Elahi D, Tobin JD, Muller DC, Brant L. Impact of age on weight goals. *Ann Intern Med*. 1985;103(6 (Pt 2)):1030-1033.

44. Heiat A. Impact of Age on Definition of Standards for Ideal Weight. *Prev Cardiol*. 2003;6(2):104-107. doi:10.1111/j.1520-037X.2003.01046.x

45. DeCaria JE, Sharp C, Petrella RJ. Scoping review report: obesity in older adults. *Int J Obes*. March 2012. doi:10.1038/ijo.2012.29

For peer review only

Funding

This work was supported by Cancer Research UK (C1417/A22962) and the ESRC (ES/R005990/1). The funders had no final role in the study design; in the collection, analysis and interpretation of data; in the writing of the report; or in the decision to submit the paper for publication. All researchers listed as authors are independent from the funders and all final decisions about the research were taken by the investigators and were unrestricted. All authors had full access to all of the data (including statistical reports and tables) in the study and can take responsibility for the integrity of the data and the accuracy of the data analysis.

For peer review only

For peer review only - http://bmjopen.bmj.com/site/about/guidelines.xhtml

Acknowledgments

Authors' contributions

SEJ analysed the data and drafted the manuscript. All authors provided critical revisions and approved the final manuscript.

Availability of data and materials

The datasets used and/or analysed during the current study are available from the corresponding author on reasonable request.

Competing interests

None.

For peer review only

Tables

Table 1 Sample demographic and anthropometric characteristics

	**Whole sample (*n*=5240)**	**Men (*n*=2352)**	**Women (*n*=2888)**	***p******
Age (mean [SD] years)	70.07 (7.76)	70.23 (7.52)	70.57 (7.71)	0.117
Ethnicity				
White	96.2	95.8	96.6	0.163
Non-white	3.8	4.2	3.4	-
SES				
Higher	32.4	38.8	26.6	<0.001
Intermediate	26.8	21.9	31.3	-
Lower	40.8	39.4	42.2	-
Self-rated health				
Good/very good/excellent	72.7	72.5	72.9	0.790
Fair/poor	27.3	27.5	27.1	-
Chronic health conditions				
None	42.0	46.2	38.1	<0.001
≥1	58.0	53.8	61.9	-
Height (mean [SD] cm)	166.31 (9.44)	173.10 (6.90)	160.01 (6.66)	<0.001
Weight (mean [SD] kg)	78.64 (16.35)	84.59 (15.04)	72.81 (15.29)	<0.001
BMI (mean [SD] kg/m^2)	28.39 (5.28)	28.21 (4.61)	28.45 (5.76)	0.116
Weight status				
Normal-weight	27.5	23.8	30.8	<0.001
Lower normal-weight	7.1	4.7	9.2	-
Upper normal-weight	20.4	19.1	21.5	-
Overweight	40.3	46.1	35.0	-
Obese	32.3	30.1	34.2	-

Weighted means and proportions are shown. Sample sizes (*n*) are shown unweighted.
**p* values are for the association between each variable and sex.
SD = standard deviation; SES = socioeconomic status; BMI = body mass index.

For peer review only

Table 2 Multivariable models testing associations with feeling 'too heavy' and 'too light' among normal-weight older men and women

	Too heavy						Too light					
	Men (*n*=581)			Women (*n*=917)			Men (*n*=581)			Women (*n*=917)		
	%*	OR [95% CI]	*p*	%*	OR [95% CI]	*p*	%*	OR [95% CI]	*p*	%*	OR [95% CI]	*p*
Age (years)	-	1.01 [0.96-1.06]	0.717	-	0.97 [0.94-1.00]	0.049	-	1.01 [0.97-1.05]	0.745	-	1.04 [1.00-1.09]	0.042
Ethnicity												
White	5.7	1.00	-	12.6	1.00	-	12.2	1.00	-	7.1	1.00	-
Non-white	4.8	0.94 [0.14-6.55]	0.952	9.5	0.42 [0.09-1.89]	0.258	42.9	6.26 [2.09-18.76]	0.001	9.5	1.96 [0.36-10.82]	0.440
SES												
Higher	5.4	1.00	-	11.9	1.00	-	10.3	1.00	-	3.7	1.00	-
Intermediate	7.8	1.40 [0.53-3.71]	0.504	12.5	1.18 [0.64-2.17]	0.605	10.7	0.84 [0.36-1.96]	0.689	9.3	2.26 [0.87-5.86]	0.093
Lower	5.1	1.03 [0.40-2.70]	0.948	13.4	1.26 [0.69-2.31]	0.457	19.7	1.30 [0.64-2.64]	0.474	8.9	2.15 [0.83-5.57]	0.114
Self-rated health												
Good/very good/excellent	5.6	1.00	-	13.1	1.00	-	8.2	1.00	-	4.8	1.00	-
Fair/poor	5.8	1.17 [0.47-2.92]	0.730	9.9	0.88 [0.45-1.72]	0.717	29.2	3.70 [1.88-7.28]	<0.001	17.6	2.61 [1.27-5.35]	0.009
BMI	-	1.76 [1.18-2.61]	0.005	-	2.14 [1.69-2.72]	<0.001	-	0.53 [0.43-0.64]	<0.001	-	0.47 [0.38-0.58]	<0.001
Chronic health conditions												
None	-	-	-	-	-	-	10.0	1.00	-	4.3	1.00	-
≥1	-	-	-	-	-	-	17.3	1.07 [0.54-2.14]	0.840	10.0	2.21 [1.03-4.77]	0.043

*Indicates the percentage of normal-weight participants in each group perceiving themselves to be too heavy/too light.
OR = odds ratio; CI = confidence interval; SES = socioeconomic status; BMI = body mass index.
Weighted data. Sample sizes (*n*) are shown unweighted.

For peer review only

Table 3 Multivariable models testing associations with feeling 'about the right weight' among older men and women with overweight

	Men (*n*=1083)			Women (*n*=1052)		
	**%***	**OR [95% CI]**	***p***	**%***	**OR [95% CI]**	***p***
Age (years)	-	1.04 [1.02-1.05]	<0.001	-	1.06 [1.04-1.09]	<0.001
Ethnicity						
White	52.7	1.00	-	34.9	1.00	-
Non-white	48.8	1.63 [0.82-3.26]	0.166	61.9	3.80 [1.45-9.98]	0.007
SES						
Higher	53.5	1.00	-	28.9	1.00	-
Intermediate	54.4	1.25 [0.85-1.85]	0.258	37.5	1.79 [1.15-2.80]	0.011
Lower	50.4	1.09 [0.78-1.52]	0.606	38.1	1.73 [1.12-2.66]	0.013
Self-rated health						
Good/very good/excellent	54.3	1.00	-	34.1	1.00	-
Fair/poor	46.4	0.78 [0.55-1.10]	0.160	40.3	1.29 [0.86-1.91]	0.216
BMI	-	0.52 [0.47-0.59]	<0.001	-	0.55 [0.49-0.63]	<0.001

*Indicates the percentage of overweight participants in each group perceiving themselves to be about the right weight or too light.
OR = odds ratio; CI = confidence interval; SES = socioeconomic status.
Weighted data. Sample sizes (*n*) are shown unweighted.

For peer review only

Table 4 Multivariable models testing associations with feeling 'about the right weight' among older men and women with obesity

	Men (*n*=688)			Women (*n*=919)		
	%*	OR [95% CI]	*p*	%*	OR [95% CI]	*p*
Age (years)	-	1.04 [1.01-1.08]	0.016	-	1.04 [1.01-1.08]	0.014
Ethnicity						
White	12.7	1.00	-	8.1	1.00	-
Non-white	5.3	0.32 [0.04-2.43]	0.268	20.7	2.72 [1.02-7.27]	0.046
SES						
Higher	11.1	1.00	-	8.0	1.00	-
Intermediate	11.8	0.95 [0.46-1.97]	0.890	4.5	0.56 [0.23-1.34]	0.191
Lower	13.8	1.33 [0.73-2.41]	0.355	11.0	1.45 [0.74-2.84]	0.282
Self-rated health						
Good/very good/excellent	13.1	1.00	-	8.3	1.00	-
Fair/poor	11.3	0.93 [0.53-1.64]	0.811	9.2	0.96 [0.54-1.68]	0.876
BMI	-	0.76 [0.67-0.86]	<0.001	-	0.96 [0.90-1.03]	0.255

*Indicates the percentage of overweight participants in each group perceiving themselves to be about the right weight or too light.
OR = odds ratio; CI = confidence interval; SES = socioeconomic status.
Weighted data. Sample sizes (*n*) are shown unweighted.

For peer review only

Figure legends

Figure 1. The proportion (with 95% confidence interval) of men and women who reported feeling 'too heavy', 'about the right weight', and 'too light', by measured weight status. Weighted data shown.

For peer review only

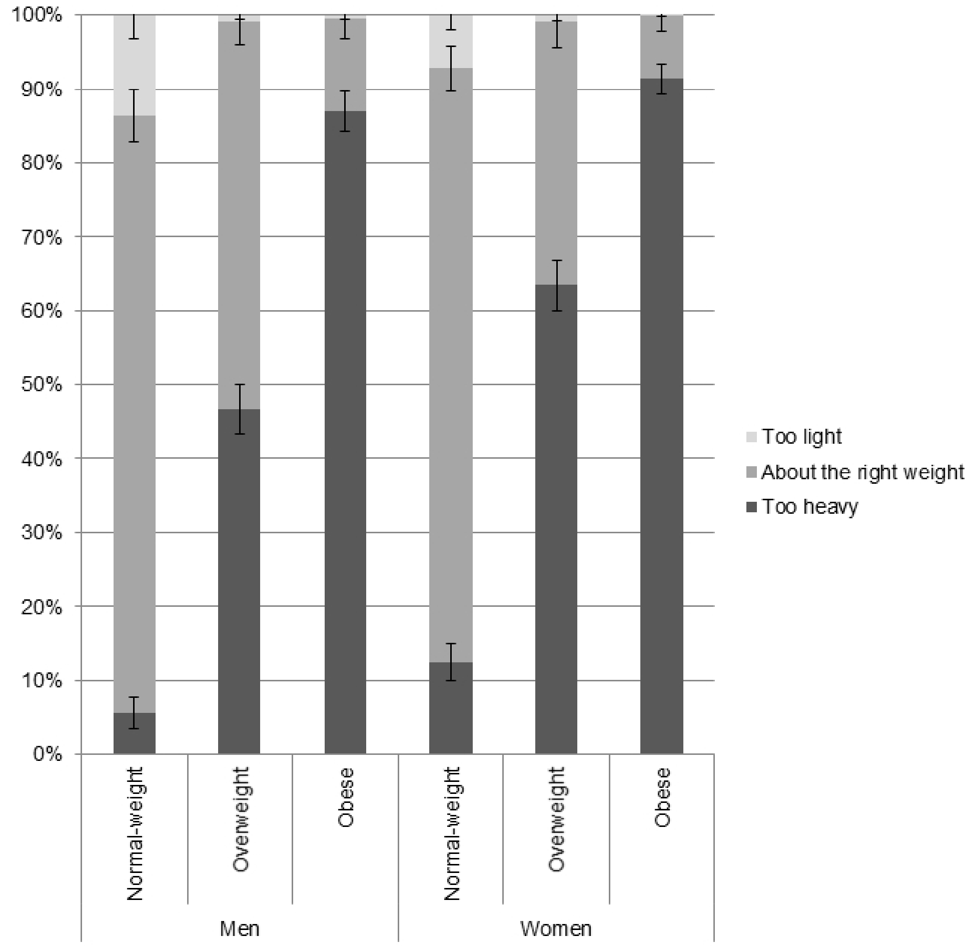

Figure 1. The proportion (with 95% confidence interval) of men and women who reported feeling 'too heavy', 'about the right weight', and 'too light', by measured weight status. Weighted data shown.

177x169mm (96 x 96 DPI)

STROBE Statement—Checklist of items that should be included in reports of ***cross-sectional studies***

	Item No	Recommendation	Page No
**Title and abstract**	1	(*a*) Indicate the study's design with a commonly used term in the title or the abstract	2
		(*b*) Provide in the abstract an informative and balanced summary of what was done and what was found	2
**Introduction**			
Background/rationale	2	Explain the scientific background and rationale for the investigation being reported	4-5
Objectives	3	State specific objectives, including any prespecified hypotheses	5
**Methods**			
Study design	4	Present key elements of study design early in the paper	5
Setting	5	Describe the setting, locations, and relevant dates, including periods of recruitment, exposure, follow-up, and data collection	5
Participants	6	(*a*) Give the eligibility criteria, and the sources and methods of selection of participants	5
Variables	7	Clearly define all outcomes, exposures, predictors, potential confounders, and effect modifiers. Give diagnostic criteria, if applicable	5-6
Data sources/ measurement	8*	For each variable of interest, give sources of data and details of methods of assessment (measurement). Describe comparability of assessment methods if there is more than one group	*5-6*
Bias	9	Describe any efforts to address potential sources of bias	6-7
Study size	10	Explain how the study size was arrived at	5
Quantitative variables	11	Explain how quantitative variables were handled in the analyses. If applicable, describe which groupings were chosen and why	5-6
Statistical methods	12	(*a*) Describe all statistical methods, including those used to control for confounding	6-7
		(*b*) Describe any methods used to examine subgroups and interactions	6-7
		(*c*) Explain how missing data were addressed	6-7
		(*d*) If applicable, describe analytical methods taking account of sampling strategy	6
		(*e*) Describe any sensitivity analyses	na
**Results**			
Participants	13*	(a) Report numbers of individuals at each stage of study—eg numbers potentially eligible, examined for eligibility, confirmed eligible, included in the study, completing follow-up, and analysed	5
		(b) Give reasons for non-participation at each stage	Na
		(c) Consider use of a flow diagram	Na
Descriptive data	14*	(a) Give characteristics of study participants (eg demographic, clinical, social) and information on exposures and potential confounders	7
		(b) Indicate number of participants with missing data for each variable of interest	Tables
Outcome data	15*	Report numbers of outcome events or summary measures	Tables
Main results	16	(*a*) Give unadjusted estimates and, if applicable, confounder-adjusted estimates and their precision (eg, 95% confidence interval). Make clear which confounders were adjusted for and why they were included	Tables

For peer review only

For peer review only - http://bmjopen.bmj.com/site/about/guidelines.xhtml

		(*b*) Report category boundaries when continuous variables were categorized	Tables
		(*c*) If relevant, consider translating estimates of relative risk into absolute risk for a meaningful time period	Na
Other analyses	17	Report other analyses done—eg analyses of subgroups and interactions, and sensitivity analyses	na
**Discussion**			
Key results	18	Summarise key results with reference to study objectives	9-10
Limitations	19	Discuss limitations of the study, taking into account sources of potential bias or imprecision. Discuss both direction and magnitude of any potential bias	11
Interpretation	20	Give a cautious overall interpretation of results considering objectives, limitations, multiplicity of analyses, results from similar studies, and other relevant evidence	11-12
Generalisability	21	Discuss the generalisability (external validity) of the study results	11
**Other information**			
Funding	22	Give the source of funding and the role of the funders for the present study and, if applicable, for the original study on which the present article is based	17

*Give information separately for exposed and unexposed groups.

Note: An Explanation and Elaboration article discusses each checklist item and gives methodological background and published examples of transparent reporting. The STROBE checklist is best used in conjunction with this article (freely available on the Web sites of PLoS Medicine at http://www.plosmedicine.org/, Annals of Internal Medicine at http://www.annals.org/, and Epidemiology at http://www.epidem.com/). Information on the STROBE Initiative is available at www.strobe-statement.org.

For peer review only

BMJ Open

Weight perceptions in older adults: findings from the English Longitudinal Study of Ageing

Journal:	*BMJ Open*
Manuscript ID	bmjopen-2019-033773.R1
Article Type:	Original research
Date Submitted by the Author:	11-Dec-2019
Complete List of Authors:	Jackson, Sarah ; University College London, Behavioural Science and Health<br>Smith, Lee; Anglia Ruskin University - Cambridge Campus, Department of Life Sciences<br>Steptoe, Andrew; University College London, Epidemiology and Public Health
**Primary Subject Heading**:	Public health
Secondary Subject Heading:	Geriatric medicine
Keywords:	GERIATRIC MEDICINE, PUBLIC HEALTH, EPIDEMIOLOGY

SCHOLARONE™
Manuscripts

I, the Submitting Author has the right to grant and does grant on behalf of all authors of the Work (as defined in the below author licence), an exclusive licence and/or a non-exclusive licence for contributions from authors who are: i) UK Crown employees; ii) where BMJ has agreed a CC-BY licence shall apply, and/or iii) in accordance with the terms applicable for US Federal Government officers or employees acting as part of their official duties; on a worldwide, perpetual, irrevocable, royalty-free basis to BMJ Publishing Group Ltd ("BMJ") its licensees and where the relevant Journal is co-owned by BMJ to the co-owners of the Journal, to publish the Work in this journal and any other BMJ products and to exploit all rights, as set out in our licence.

The Submitting Author accepts and understands that any supply made under these terms is made by BMJ to the Submitting Author unless you are acting as an employee on behalf of your employer or a postgraduate student of an affiliated institution which is paying any applicable article publishing charge ("APC") for Open Access articles. Where the Submitting Author wishes to make the Work available on an Open Access basis (and intends to pay the relevant APC), the terms of reuse of such Open Access shall be governed by a Creative Commons licence – details of these licences and which Creative Commons licence will apply to this Work are set out in our licence referred to above.

Other than as permitted in any relevant BMJ Author's Self Archiving Policies, I confirm this Work has not been accepted for publication elsewhere, is not being considered for publication elsewhere and does not duplicate material already published. I confirm all authors consent to publication of this Work and authorise the granting of this licence.

For peer review only

Weight perceptions in older adults: findings from the English Longitudinal Study of Ageing

Sarah E. Jackson[1], Lee Smith[2], Andrew Steptoe[1]

[1]Department of Behavioural Science and Health, University College London, London, UK

[2] Cambridge Centre for Sport and Exercise Sciences, Anglia Ruskin University, Cambridge, UK

Corresponding author: Sarah E Jackson, PhD. Department of Behavioural Science and Health, University College London, 1-19 Torrington Place, London WC1E 6BT

Tel: (44) 207 679 3179

Fax: (44) 207 916 8354

s.e.jackson@ucl.ac.uk

Word count: 3,465

For peer review only

For peer review only - http://bmjopen.bmj.com/site/about/guidelines.xhtml

Abstract

Objectives: To explore weight perceptions in a large, nationally-representative sample of older adults, and the extent to which they differ according to age and perceived health status.

Setting: England.

Participants: 5,240 men and women (≥50y) participating in the English Longitudinal Study of Ageing (2016/17).

Main outcome measures: Weight perception was self-reported as too heavy, too light, or about right.

Results: The majority of older adults endorsed a weight perception that matched their (objectively measured) BMI classification. However, one in ten (9.9%) older adults classified by BMI as normal-weight (18.5-24.9kg/m^2) felt too light, with women at the upper end of the older age spectrum (OR=1.04, 95%CI=1.01-1.09), and men (OR=3.70, 95%CI=1.88-7.28) and women (OR=2.61, 95%CI=1.27-5.35) in poorer health more likely to do so. Almost half (44.8%) of older adults classified as overweight (25-29.9kg/m^2) and one in ten (10.3%) classified as obese (≥30kg/m^2) felt about the right weight, with this observed more frequently among men and women at the upper end of the older age spectrum (OR range 1.04-1.06).

Conclusion: Older adults' perceptions of their own weight generally correspond with traditional BMI cut-offs for normal-weight, overweight, and obesity. However, a substantial minority 'underestimate' their weight status, with those at the upper end of the age spectrum and those in poorer health more likely to do so.

Key words: body mass index; weight perceptions; population-based study; obesity paradox

For peer review only - http://bmjopen.bmj.com/site/about/guidelines.xhtml

Strengths and limitations of the study

Data were from a large, representative sample of older adults in England.

Height and weight were objectively measured for calculation of body mass index.

However, there was a substantial amount of missing data, so findings may not generalise to the entire population.

If those who were more concerned about their weight were more likely to decline to be measured, our results may underestimate the proportion of older adults across all weight groups who consider themselves to be too heavy.

For peer review only

For peer review only - http://bmjopen.bmj.com/site/about/guidelines.xhtml

Introduction

The global obesity epidemic and ageing population are major public health concerns. With excess weight becoming increasingly 'normal', public perceptions of what constitutes a healthy body weight have become more inaccurate over time, with increasing numbers perceiving a body mass index (BMI) in the overweight or obese range (≥25 kg/m^2) to be 'about right' [1]. The numbers of older adults with overweight (BMI 25-29.9 kg/m^2) and obesity (BMI ≥30 kg/m^2) are rising rapidly, due to concurrent increases in the number of adults who reach older age and the proportion who carry excess weight [2–4]. Understanding how older adults perceive their own weight status, and the extent to which this is influenced by their age and health status, is important for informing targeted recommendations and interventions to promote healthy weight in later life.

Older adults' weight perceptions may be influenced by several social and physiological factors. On a societal level, there are strong preferences for slimness in women and lean muscularity in men [5]. Some evidence suggests that older people may be less influenced by, and feel less pressure to attain, cultural body weight ideals [6,7]. Nonetheless, body dissatisfaction is evident in mid- and later life [8–12] and may even increase as age-related changes in body composition widen the discrepancy between ideal and actual body image [13]. Ageing is associated with an increase in fat mass, loss of lean muscle mass, and redistribution of adipose tissue to the abdominal region [14]; changes that may occur without concomitant changes in body weight and BMI [15].

While few previous studies have examined older adults' weight perceptions, those that have indicate age-related changes in perceptions of body weight in relation to actual BMI category. For example, in a sample of adult Korean women (*n*=8,906, age 20-79 years), older women were more likely than younger women to underestimate their weight status relative to their actual BMI category (50.7% of 70-79 year olds vs. 12.6% of 20-29 year olds) and less likely to overestimate their weight status (7.4% vs. 18.7%, respectively) [16]. Similarly, in a study of older Dutch men and women (*n*=1,295, age 60-96 years), the proportion of women who underestimated their weight increased with age (OR=2.97, 95% CI 1.59-5.57 for 80-96 year olds vs. 60-69 year olds), although no such pattern was found for men [17]. However, it was not clear from these studies whether these differences are driven by differences between older and younger people, for example relating to health status. Qualitative research suggests at least some older adults believe carrying extra weight could be protective in times of illness [12], which may mean older people's weight perceptions are influenced by current perceptions of health status or future health concerns.

For peer review only - http://bmjopen.bmj.com/site/about/guidelines.xhtml

The present study therefore aimed to explore perceptions of weight in a large, nationally-representative sample of older adults living in England, and the extent to which they differ according to age and perceived health status.

Method

Study population

Data were from the English Longitudinal Study of Ageing (ELSA), a panel study of men and women aged ≥50 years living in England. Full details of the study's sampling procedure and methods are available elsewhere [18]. The present analyses use data collected in the eighth wave of ELSA (collected 2016/17), as this is the only wave in which weight perceptions have been assessed. Of the 8,445 participants interviewed, 5,240 (62.0%) had complete data on all variables of interest and comprised our analytic sample.

Measures

Weight perception

Weight perception was assessed with the question "*Given your age and height, would you say that you are about the right weight, too heavy, or too light?*".

Anthropometric data

Weight was measured by nurses to the nearest 0.1 kg using portable electronic scales. Height was measured in Wave 6 (it was not included in the Wave 8 assessment) to the nearest millimetre using a portable stadiometer. Nurses recorded any factors that might have compromised the reliability of the measurements (e.g. participant was stooped/unwilling to remove shoes) and these cases were excluded. BMI was calculated as weight in kilograms divided by the square of height in metres, and categorised as underweight (<18.5 kg/m^2), normal-weight (18.5 to <25 kg/m^2), overweight (25 to <30 kg/m^2) and obese (≥30 kg/m^2). We excluded participants with a BMI in the underweight range because there were insufficient numbers for meaningful analysis as a separate group (*n*=64, 1.2% of otherwise eligible sample).

For some descriptive analyses, normal-weight participants were divided into those with a BMI in the lower half of the normal-weight range (BMI 18.5 to <21.75 kg/m^2; 'lower normal-weight') and those in the upper

For peer review only - http://bmjopen.bmj.com/site/about/guidelines.xhtml

half (BMI 21.75 to <30 kg/m^2; 'upper normal-weight') to provide an indication as to the distribution of participants across the normal-weight range and help interpret associations between BMI and weight perceptions.

Sociodemographic information

Information on age, sex, ethnicity (white vs. non-white), and SES was recorded. SES was indexed using the short version of the NSSEC 3-category classification of the present or most recent occupation and categorised as higher (managerial/professional occupations), intermediate (intermediate occupations), and lower (routine/manual occupations) [19]. This measure of SES was chosen for comparability with previous studies investigating weight perceptions in other age groups in England [e.g. 20].

Perceived health and comorbidities

Self-rated health was assessed using a single item: *"Would you say your health is… poor/fair/good/very good/excellent?"* We analysed the proportion of individuals rating their health as fair/poor. This dichotomy is commonly used in analyses of this variable [21–23] to overcome issues relating to the skewed distribution of responses and provide results that are easily interpreted (i.e. odds of the outcome associated with poorer versus better health).

Information about five doctor-diagnosed chronic diseases that may cause weight loss (cancer, stroke, chronic lung disease, diabetes, and arthritis) was self-reported and the number of reported conditions were summed to create a chronic health condition index ranging from 0-5. Because scores were highly skewed, we dichotomised this variable to distinguish between 0 and ≥1 health conditions.

Statistical analysis

Analyses were performed using IBM SPSS Statistics 24. In order to produce representative estimates for older adults in the English population, data were weighted to correct for sampling probabilities and to match the English population on age and sex. The weights accounted for the differential probability of being included in Wave 8 of ELSA.

Analyses were performed separately for men and women. We tested sex differences in sociodemographic, anthropometric, self-rated health, and weight perception variables using *t*-tests for continuous variables and Pearson's chi-square analyses for categorical variables. We used multivariable logistic regression to

For peer review only - http://bmjopen.bmj.com/site/about/guidelines.xhtml

For peer review only

identify independent associations with perception of weight as (i) 'too heavy' or (ii) 'too light' among normal-weight participants, and associations with perception of weight as 'about right' among participants with overweight or obesity. Variables included in the models were age, ethnicity, SES, self-rated health, and BMI. In order to evaluate whether perception of weight as too light among normal-weight participants, or about right among participants with overweight or obesity, was associated with health conditions that may cause weight loss, we also adjusted for chronic health conditions in each model. Results are reported as odds ratios (ORs) with 95% confidence intervals (CIs).

Patient and public involvement

Patients and the public were not involved in this research.

Results

There were 2,352 men and 2,888 women in the sample. Descriptive characteristics overall and by sex are shown in Table 1. The mean age was 70.1 (SD 7.8) years. The majority of participants were white (96.2%) and rated their health as good, very good, or excellent (72.7%) despite many having one or more chronic health conditions (58.0%). There was fairly even distribution across socioeconomic groups (32.4% higher SES, 26.8% intermediate SES, 40.8% lower SES), although women were less likely than men to be in the higher SES group (26.6% vs. 38.8%, $p<0.001$). Just over a quarter (27.5%) of participants had a BMI in the normal-weight range, 40.3% were overweight, and a further 32.3% had obesity. On average, men were significantly taller and heavier than women ($p<0.001$). While there was no significant sex difference in mean BMI ($p=0.116$), men were more likely than women to have a BMI placing them in the overweight range (46.1% vs. 35.0%) while women were more likely than men to have a normal-weight (30.8% vs. 23.8%) or obese BMI (34.2% vs. 30.1%, $p<0.001$).

Weight perception among normal-weight older adults

Figure 1 summarises the distribution of weight perceptions by sex and weight status. In the normal-weight category, the majority (80.3%) of participants thought they were about the right weight, but 9.9% thought they were too light, and 9.7% thought they were too heavy. Normal-weight women were significantly more likely than normal-weight men to consider themselves to be too heavy (12.6% vs. 5.6%, $p<0.001$) and less likely to consider themselves too light (7.3% vs. 13.7%, $p<0.001$).

For peer review only - http://bmjopen.bmj.com/site/about/guidelines.xhtml

Multivariable models testing independent associations of age, sex, ethnicity, SES, and BMI with perception of body weight as too heavy in normal-weight men and women are presented in Table 2 (left panel). There was a significant independent association between BMI and perception of weight as too heavy in both sexes, with each unit increase in BMI associated with 1.76 times higher odds (95% CI 1.18-2.61) of perception of weight as too heavy in men and 2.14 times higher odds (95% CI 1.69-2.72) in women. Some 6.7% of men and 16.8% of women in the upper normal-weight group felt too heavy, compared with just 1.1% of men and 2.5% of women in the lower normal-weight group. Older age was significantly associated with reduced odds of perception of weight as too heavy in women (OR=0.97, 95% CI 0.94-1.00) but not in men. There was no significant independent association between perception of weight as too heavy and ethnicity, SES, or self-rated health in either sex.

Factors independently associated with perception of body weight as too light in normal-weight men and women are shown in Table 2 (right panel). There were strong independent associations with BMI and self-rated health. In both sexes, odds of feeling too light were significantly lower among those with a higher BMI, with each unit increase in BMI associated with a 47% reduction in odds in men (OR=0.53, 95% CI 0.43-0.64) and a 53% reduction in odds in women (OR=0.47, 95% CI 0.38-0.58). Just 6.5% of men and 2.6% of women in the upper normal-weight group felt too light, compared with 43.3% of men and 17.8% of women in the lower normal-weight group. Odds of perception of weight as too light were significantly increased among participants with fair/poor self-rated health (men: OR=3.70, 95% CI 1.88-7.28; women: OR=2.61, 95% CI 1.27-5.35). The presence of one or more chronic health conditions was also independently associated with increased odds of perception of weight as too light in women (OR=2.21, 95% CI 1.03-4.77) but not in men. Older age was associated with increased odds of perception of weight as too light in normal-weight women (OR=1.04, 95% CI 1.01-1.09) but not in men. Non-white ethnicity was associated with increased odds of perception of weight as too light in normal-weight men (OR=6.26, 95% CI 2.09-18.76) but not in women. There was no significant independent association with SES in either sex.

Weight perception among older adults with overweight or obesity

Among participants with a BMI in the overweight range, 54.3% thought they were too heavy, but 44.8% thought they were about the right weight and 0.9% thought they were too light. Among participants with an obese BMI, 89.4% thought they were too heavy, 10.3% thought they were about the right weight and 0.4% thought they were too light. Women with overweight were more likely than men with overweight to recognise that they were too heavy (63.4% vs. 46.7%, p<0.001) and less likely to perceive themselves to be about the right weight (35.7% vs. 52.5%, p<.001). Likewise, women with obesity were more likely than men

For peer review only - http://bmjopen.bmj.com/site/about/guidelines.xhtml

with obesity to perceive their weight as too heavy (91.3% vs. 87.0%, *p*=0.012) and less likely to perceive their weight as about right (8.5% vs. 12.4%, *p*=0.020) (Figure 1).

Factors independently associated with perception of body weight as about right in men and women with overweight are summarised in Table 3. Among overweight men and women, there was a strong independent association with BMI: each unit increase in BMI was associated with 48% lower odds of perception of weight as about right in men (OR=0.52, 95% CI 0.47-0.59) and 45% lower odds in women (OR=0.55, 95% CI 0.48-0.63). Older age was also independently associated with increased odds of perception of weight as about right in both sexes (men: OR=1.04, 95% CI 1.02-1.06; women: OR=1.06, 95% CI 1.04-1.09). Non-white ethnicity was associated with increased odds of perception of weight as about right in women (OR=3.79, 95% CI 1.44-9.94) but not in men. An association with SES was also observed in women, with women with overweight in the intermediate and lower SES groups significantly more likely to perceive their weight to be about right than those from the higher SES group (intermediate: OR=1.78, 95% CI 1.14-2.79; lower: OR=1.73, 95% CI 1.13-2.66). There was no significant association with SES in men. There was no significant independent association with self-rated health in either sex, although in men (but not in women), presence of at least one comorbid health condition was associated with significantly lower odds of perception of weight as about right (OR=0.56, 95% CI 0.41-0.76).

Factors independently associated with perception of body weight as about right in men and women with obesity are shown in Table 4. Older age was significantly associated with increased odds of perception of body weight as about right in both men (OR=1.05, 95% CI 1.01-1.09) and women (OR=1.04, 95% CI 1.01-1.08). Higher BMI was associated with 24% lower odds of perception of body weight as about right in men (OR=0.76, 95% CI 0.67-0.87) but no significant association with BMI was observed in women. Non-white ethnicity was significantly associated with increased odds of perception of body weight as about right in women (OR=2.76, 95% CI 1.03-7.40) but not in men. No significant association between perception of body weight as about right and SES, self-rated health, or chronic health conditions was observed in either sex.

Discussion

In a large, representative sample of older adults living in England, we found that weight perceptions broadly corresponded to participants' actual weight status as defined by widely used BMI cut-offs: 80% of older adults with a BMI in the normal-weight range thought they were about the right weight, and over 50% of

For peer review only - http://bmjopen.bmj.com/site/about/guidelines.xhtml

older adults with a BMI in the overweight range and almost 90% of those with a BMI in the obese range thought they were too heavy. However, a substantial number of older adults either under- or overestimated their weight status relative to their BMI category. One in ten participants with a normal-weight BMI thought they were too heavy and one in ten thought they were too light. Almost half of participants with an overweight BMI thought they were about the right weight, as did one in ten of those with an obese BMI.

As has been observed in previous research examining weight perceptions in younger samples [1,20,24], there were systematic differences between men and women's weight perceptions. Across all BMI categories, men were consistently more likely than women to underestimate their weight status, and normal-weight women were more likely than normal-weight men to report feeling too heavy. Within BMI categories, those with a higher BMI were more likely to perceive themselves to be too heavy and less likely to perceive themselves to be too light or about right. There were also some differences in weight perceptions by ethnicity and SES, with people from non-white ethnic groups and intermediate and lower SES groups more likely to underestimate their weight status, as has been shown previously in younger samples [20,24], although these differences were not consistently observed across BMI categories or sexes.

Importantly, there were also clear age-related differences in weight perceptions across all BMI categories. In men and women with an overweight or obese BMI, the odds of feeling about the right weight increased with advancing age. In women with a normal-weight BMI, the odds of feeling too light increased and the odds of feeling too heavy decreased with age, although there were no significant differences by age in men. These findings are suggestive of a higher ideal weight at older ages. This is consistent with previous studies that have shown people, particularly women, tend to endorse a slightly larger and more curvaceous body shape as they get older [8,25]. It is possible that older people believe that having an overweight or obese BMI is not necessarily a bad thing at older ages [12]. Alternatively, as people get older they may give up the effort to reduce weight at higher BMIs, perhaps because other health issues become more salient for them, or because they are less interested in self-presentation and striving for a slimmer physique [12].

There was also a significant association between self-rated health and odds of feeling too light among men and women with a normal-weight BMI. Those who rated their health as fair or poor had around three times higher odds of feeling too light at a normal-weight BMI than those who rated their health as good, very good, or excellent. In addition, women with a normal-weight BMI who had at least one chronic condition were also more likely to consider themselves to be too light, although there was no difference in men. These results could be interpreted as suggesting that the ideal weight may be higher for older adults in

For peer review only - http://bmjopen.bmj.com/site/about/guidelines.xhtml

poorer health than for those in good health. There is a vast literature documenting an obesity paradox in chronic conditions including cardiovascular disease [26], cancer [27], kidney disease [28], and lung disease [29], whereby patients who carry excess weight have better outcomes than those with a normal-weight BMI. It has also been suggested that stress related to negative body image perception may have a causal role in the development of poor health [30]. An alternative explanation is that the association between poorer self-rated health and increased likelihood of feeling too light might be secondary to illness. Older people with serious chronic illnesses often lose weight, so the fact they think they are too light might be a reflection of this concern.

At the upper end of the weight spectrum, health status was less strongly associated with weight perceptions, with no significant association observed between self-rated health and perception of weight as about right among those with an overweight or obese BMI. However, men with a BMI in the overweight range who had at least one comorbid condition had significantly lower odds of feeling their weight was about right than those who were free of comorbidities, suggesting that experiencing an associated health problem may make men more likely to recognise their overweight (albeit still less likely than women with or without a health condition).

Taken together, these findings suggest that the discrepancy between perceived weight status and BMI-based definitions of weight status is greater among people who are older or in poorer health. The appropriateness of conventional BMI definitions of weight status for older people is an issue of ongoing debate in the literature [31–35]. Currently, guidance issued by official bodies such as the UK National Health Service [36] and US Centers for Disease Control and Prevention [37] does not differ according to age group or health status. However, many health professionals are reluctant to recommend weight loss for older patients with an overweight BMI [38,39] and there have been calls to reconsider the standards for ideal weight at older ages and develop age-specific recommendations [40–42]. The present results show that for the majority of older people, weight perceptions map onto BMI definitions of weight status. Further research is needed to establish whether for the remainder, the mismatch between perceived weight and BMI status represents a lack of awareness of 'healthy' weight, preference for higher weight in older age, or reflects a genuine biological advantage to being heavier in older age (the so-called 'obesity paradox' [35]).

Strengths of the present study include the large sample size and objective measurements of height and weight. However, findings should be considered in the light of several limitations. There was a substantial amount of missing data, so findings may not generalise to the entire population. Of note, weight

For peer review only - http://bmjopen.bmj.com/site/about/guidelines.xhtml

measurements were not available for all Wave 8 participants (8% missing). If those who were more concerned about their weight were more likely to decline to be measured, our results may underestimate the proportion of older adults across all weight groups who consider themselves to be too heavy. In addition, no data were collected on participants' weight history, which could potentially influence their current perceptions of their body weight (for example, individuals with a higher average lifetime BMI or history of overweight or obesity may be more likely to incorrectly perceive themselves to be 'too light' or 'about right' in older age).

In conclusion, the present results indicate that older adults' perceptions of their own weight generally correspond with traditional BMI cut-offs for normal-weight, overweight, and obesity. However, a substantial minority 'underestimate' their weight status, with those at the upper end of the age spectrum and those in poorer health more likely to do so.

For peer review only - http://bmjopen.bmj.com/site/about/guidelines.xhtml

References

1. Johnson F, Cooke L, Croker H, Wardle J. Changing perceptions of weight in Great Britain: comparison of two population surveys. *BMJ*. 2008;337(jul10 1):a494-a494. doi:10.1136/bmj.a494

2. Vincent GK, Velkoff VA. *The next Four Decades: The Older Population in the United States: 2010 to 2050*. US Department of Commerce, Economics and Statistics Administration, US Census Bureau; 2010.

3. Wang YC, McPherson K, Marsh T, Gortmaker SL, Brown M. Health and economic burden of the projected obesity trends in the USA and the UK. *The Lancet*. 2011;378(9793):815-825. doi:10.1016/S0140-6736(11)60814-3

4. Villareal DT, Apovian CM, Kushner RF, Klein S. Obesity in Older Adults: Technical Review and Position Statement of the American Society for Nutrition and NAASO, The Obesity Society. *Obes Res*. 2005;13(11):1849-1863. doi:10.1038/oby.2005.228

5. Swami V. Cultural Influences on Body Size Ideals. *Eur Psychol*. 2015;20(1):44-51. doi:10.1027/1016-9040/a000150

6. Bedford JL, Johnson CS. Societal Influences on Body Image Dissatisfaction in Younger and Older Women. *J Women Aging*. 2006;18(1):41-55. doi:10.1300/J074v18n01_04

7. Dunkel TM, Davidson D, Qurashi S. Body satisfaction and pressure to be thin in younger and older Muslim and non-Muslim women: The role of Western and non-Western dress preferences. *Body Image*. 2010;7(1):56-65. doi:10.1016/j.bodyim.2009.10.003

8. Tiggemann M. Body image across the adult life span: stability and change. *Body Image*. 2004;1(1):29-41. doi:10.1016/S1740-1445(03)00002-0

9. Webster J, Tiggemann M. The Relationship Between Women's Body Satisfaction and Self-Image Across the Life Span: The Role of Cognitive Control. *J Genet Psychol*. 2003;164(2):241-252. doi:10.1080/00221320309597980

10. Lamb CS, Jackson LA, Cassiday PB, Priest DJ. Body figure preferences of men and women: A comparison of two generations. *Sex Roles*. 1993;28(5-6):345-358. doi:10.1007/BF00289890

11. Rozin P, Fallon A. Body image, attitudes to weight, and misperceptions of figure preferences of the opposite sex: a comparison of men and women in two generations. *J Abnorm Psychol*. 1988;97(3):342-345.

12. Jackson SE, Holter L, Beeken RJ. 'Just because I'm old it doesn't mean I have to be fat': a qualitative study exploring older adults' views and experiences of weight management. *BMJ Open*. 2019;9(2):e025680. doi:10.1136/bmjopen-2018-025680

13. Tiggemann M. Body-size dissatisfaction: Individual differences in age and gender, and relationship with self-esteem. *Personal Individ Differ*. 1992;13(1):39-43. doi:10.1016/0191-8869(92)90215-B

For peer review only - http://bmjopen.bmj.com/site/about/guidelines.xhtml

14. St-Onge M-P, Gallagher D. Body composition changes with aging: The cause or the result of alterations in metabolic rate and macronutrient oxidation? *Nutr Burbank Los Angel Cty Calif*. 2010;26(2):152-155. doi:10.1016/j.nut.2009.07.004

15. St-Onge M-P. Relationship between body composition changes and changes in physical function and metabolic risk factors in aging. *Curr Opin Clin Nutr Metab Care*. 2005;8(5):523-528.

16. Park B, Cho HN, Choi E, et al. Self-perceptions of body weight status according to age-groups among Korean women: A nationwide population-based survey. *PLOS ONE*. 2019;14(1):e0210486. doi:10.1371/journal.pone.0210486

17. Monteagudo Sánchez C, Dijkstra SC, Visser M. Self-perception of body weight status in older Dutch adults. *J Nutr Health Aging*. 2015;19(6):612-618. doi:10.1007/s12603-015-0486-z

18. Steptoe A, Breeze E, Banks J, Nazroo J. Cohort profile: the English Longitudinal Study of Ageing. *Int J Epidemiol*. 2013;42(6):1640-1648. doi:10.1093/ije/dys168

19. Rose D, Pevalin DJ. *A Researcher's Guide to the National Statistics Socio-Economic Classification*. SAGE Publications Ltd; 2003.

20. Jackson SE, Johnson F, Croker H, Wardle J. Weight perceptions in a population sample of English adolescents: cause for celebration or concern? *Int J Obes*. 2015;39(10):1488-1493. doi:10.1038/ijo.2015.126

21. Steptoe A, Jackson SE. The Life Skills of Older Americans: Association with Economic, Psychological, Social, and Health Outcomes. *Sci Rep*. 2018;8(1):9669. doi:10.1038/s41598-018-27909-w

22. DeSalvo KB, Bloser N, Reynolds K, He J, Muntner P. Mortality prediction with a single general self-rated health question. A meta-analysis. *J Gen Intern Med*. 2006;21(3):267-275. doi:10.1111/j.1525-1497.2005.00291.x

23. Jackson SE, Hackett RA, Steptoe A. Associations between age discrimination and health and wellbeing: cross-sectional and prospective analysis of the English Longitudinal Study of Ageing. *Lancet Public Health*. 2019;in press.

24. Yaemsiri S, Slining MM, Agarwal SK. Perceived weight status, overweight diagnosis, and weight control among US adults: the NHANES 2003–2008 Study. *Int J Obes*. 2011;35(8):1063-1070. doi:10.1038/ijo.2010.229

25. Clarke LCH. Beauty in Later Life: Older Women's Perceptions of Physical Attractiveness. *Can J Aging Rev Can Vieil*. 2002;21(3):429-442. doi:10.1017/S0714980800001744

26. Lavie CJ, Milani RV, Ventura HO. Obesity and the "Obesity Paradox" in Cardiovascular Diseases. *Clin Pharmacol Ther*. 2011;90(1):23-25. doi:10.1038/clpt.2011.87

27. Lennon H, Sperrin M, Badrick E, Renehan AG. The Obesity Paradox in Cancer: a Review. *Curr Oncol Rep*. 2016;18(9):56. doi:10.1007/s11912-016-0539-4

For peer review only

For peer review only - http://bmjopen.bmj.com/site/about/guidelines.xhtml

28. Park J, Ahmadi S-F, Streja E, et al. Obesity Paradox in End-Stage Kidney Disease Patients. *Prog Cardiovasc Dis*. 2014;56(4):415-425. doi:10.1016/j.pcad.2013.10.005

29. Chittal P, Babu AS, Lavie CJ. Obesity Paradox: Does Fat Alter Outcomes in Chronic Obstructive Pulmonary Disease? *COPD J Chronic Obstr Pulm Dis*. 2015;12(1):14-18. doi:10.3109/15412555.2014.915934

30. Muennig P, Jia H, Lee R, Lubetkin E. I Think Therefore I Am: Perceived Ideal Weight as a Determinant of Health. *Am J Public Health*. 2008;98(3):501-506. doi:10.2105/AJPH.2007.114769

31. Beck AM, Ovesen L. At which body mass index and degree of weight loss should hospitalized elderly patients be considered at nutritional risk? *Clin Nutr Edinb Scotl*. 1998;17(5):195-198.

32. Sardinha LB, Teixeira PJ. Obesity screening in older women with the body mass index: A receiver operating characteristic (ROC) analysis. *Sci Sports*. 2000;15(4):212-219. doi:10.1016/S0765-1597(00)80008-8

33. Cook Z, Kirk S, Lawrenson S, Sandford S. Use of BMI in the assessment of undernutrition in older subjects: reflecting on practice. *Proc Nutr Soc*. 2005;64(3):313-317. doi:10.1079/PNS2005437

34. Vasconcelos F de AG de, Cordeiro BA, Rech CR, Petroski EL. Sensitivity and specificity of the body mass index for the diagnosis of overweight/obesity in elderly. *Cad Saúde Pública*. 2010;26:1519-1527. doi:10.1590/S0102-311X2010000800006

35. Oreopoulos A, Kalantar-Zadeh K, Sharma AM, Fonarow GC. The Obesity Paradox in the Elderly: Potential Mechanisms and Clinical Implications. *Clin Geriatr Med*. 2009;25(4):643-659. doi:10.1016/j.cger.2009.07.005

36. NHS Digital. Healthy weight. nhs.uk. https://www.nhs.uk/live-well/healthy-weight/. Published March 17, 2019. Accessed March 17, 2019.

37. Centers for Disease Control and Prevention. Assessing Your Weight | Healthy Weight. https://www.cdc.gov/healthyweight/assessing/index.html. Published March 6, 2019. Accessed March 17, 2019.

38. Houston DK, Nicklas BJ, Zizza CA. Weighty Concerns: The Growing Prevalence of Obesity among Older Adults. *J Am Diet Assoc*. 2009;109(11):1886-1895. doi:10.1016/j.jada.2009.08.014

39. Sommers A. U.S. Congressional Research Service: obesity among older Americans. 2011. http://www.legistorm.com/score_crs/show/id/104646.html. Accessed April 18, 2013.

40. Andres R, Elahi D, Tobin JD, Muller DC, Brant L. Impact of age on weight goals. *Ann Intern Med*. 1985;103(6 (Pt 2)):1030-1033.

41. Heiat A. Impact of Age on Definition of Standards for Ideal Weight. *Prev Cardiol*. 2003;6(2):104-107. doi:10.1111/j.1520-037X.2003.01046.x

42. DeCaria JE, Sharp C, Petrella RJ. Scoping review report: obesity in older adults. *Int J Obes*. March 2012. doi:10.1038/ijo.2012.29

For peer review only - http://bmjopen.bmj.com/site/about/guidelines.xhtml

For peer review only

For peer review only - http://bmjopen.bmj.com/site/about/guidelines.xhtml

Funding

This work was supported by Cancer Research UK (C1417/A22962) and the ESRC (ES/R005990/1). The funders had no final role in the study design; in the collection, analysis and interpretation of data; in the writing of the report; or in the decision to submit the paper for publication. All researchers listed as authors are independent from the funders and all final decisions about the research were taken by the investigators and were unrestricted. All authors had full access to all of the data (including statistical reports and tables) in the study and can take responsibility for the integrity of the data and the accuracy of the data analysis.

For peer review only

For peer review only - http://bmjopen.bmj.com/site/about/guidelines.xhtml

Acknowledgments

Authors' contributions

SEJ analysed the data and drafted the manuscript. LS and AS provided critical revisions and approved the final manuscript.

Availability of data and materials

The raw ELSA data are available from the UK Data Service.

Competing interests

None.

For peer review only

Tables

Table 1 Sample demographic and anthropometric characteristics

	**Whole sample (*n*=5240)**	**Men (*n*=2352)**	**Women (*n*=2888)**	***p****
Age (mean [SD] years)	70.07 (7.76)	70.23 (7.52)	70.57 (7.71)	0.117
Ethnicity				
White	96.2	95.8	96.6	0.163
Non-white	3.8	4.2	3.4	-
SES				
Higher	32.4	38.8	26.6	<0.001
Intermediate	26.8	21.9	31.3	-
Lower	40.8	39.4	42.2	-
Self-rated health				
Good/very good/excellent	72.7	72.5	72.9	0.790
Fair/poor	27.3	27.5	27.1	-
Chronic health conditions				
None	42.0	46.2	38.1	<0.001
≥1	58.0	53.8	61.9	-
Height (mean [SD] cm)	166.31 (9.44)	173.10 (6.90)	160.01 (6.66)	<0.001
Weight (mean [SD] kg)	78.64 (16.35)	84.59 (15.04)	72.81 (15.29)	<0.001
BMI (mean [SD] kg/m^2)	28.39 (5.28)	28.21 (4.61)	28.45 (5.76)	0.116
Weight status				
Normal-weight	27.5	23.8	30.8	<0.001
Lower normal-weight	7.1	4.7	9.2	-
Upper normal-weight	20.4	19.1	21.5	-
Overweight	40.3	46.1	35.0	-
Obese	32.3	30.1	34.2	-

Weighted means and proportions are shown. Sample sizes (*n*) are shown unweighted.
**p* values are for the association between each variable and sex.
SD = standard deviation; SES = socioeconomic status; BMI = body mass index.

For peer review only

Table 2 Multivariable models testing associations with feeling 'too heavy' and 'too light' among normal-weight older men and women

	Too heavy						Too light					
	Men (*n*=581)			Women (*n*=917)			Men (*n*=581)			Women (*n*=917)		
	%*	OR [95% CI]	*p*	%*	OR [95% CI]	*p*	%*	OR [95% CI]	*p*	%*	OR [95% CI]	*p*
Age (years)	-	1.01 [0.96-1.06]	0.717	-	0.97 [0.94-1.00]	0.049	-	1.01 [0.97-1.05]	0.745	-	1.04 [1.00-1.09]	0.042
Ethnicity												
White	5.7	1.00	-	12.6	1.00	-	12.2	1.00	-	7.1	1.00	-
Non-white	4.8	0.94 [0.14-6.55]	0.952	9.5	0.42 [0.09-1.89]	0.258	42.9	6.26 [2.09-18.76]	0.001	9.5	1.96 [0.36-10.82]	0.440
SES												
Higher	5.4	1.00	-	11.9	1.00	-	10.3	1.00	-	3.7	1.00	-
Intermediate	7.8	1.40 [0.53-3.71]	0.504	12.5	1.18 [0.64-2.17]	0.605	10.7	0.84 [0.36-1.96]	0.689	9.3	2.26 [0.87-5.86]	0.093
Lower	5.1	1.03 [0.40-2.70]	0.948	13.4	1.26 [0.69-2.31]	0.457	19.7	1.30 [0.64-2.64]	0.474	8.9	2.15 [0.83-5.57]	0.114
Self-rated health												
Good/very good/excellent	5.6	1.00	-	13.1	1.00	-	8.2	1.00	-	4.8	1.00	-
Fair/poor	5.8	1.17 [0.47-2.92]	0.730	9.9	0.88 [0.45-1.72]	0.717	29.2	3.70 [1.88-7.28]	<0.001	17.6	2.61 [1.27-5.35]	0.009
BMI	-	1.76 [1.18-2.61]	0.005	-	2.14 [1.69-2.72]	<0.001	-	0.53 [0.43-0.64]	<0.001	-	0.47 [0.38-0.58]	<0.001
Chronic health conditions												
None	-	-	-	-	-	-	10.0	1.00	-	4.3	1.00	-
≥1	-	-	-	-	-	-	17.3	1.07 [0.54-2.14]	0.840	10.0	2.21 [1.03-4.77]	0.043

*Indicates the percentage of normal-weight participants in each group perceiving themselves to be too heavy/too light.
OR = odds ratio; CI = confidence interval; SES = socioeconomic status; BMI = body mass index.
Weighted data. Sample sizes (*n*) are shown unweighted.

For peer review only

Table 3 Multivariable models testing associations with feeling 'about the right weight' among older men and women with overweight

	Men (*n*=1083)			Women (*n*=1052)		
	**%***	**OR [95% CI]**	***p***	**%***	**OR [95% CI]**	***p***
Age (years)	-	1.04 [1.02-1.06]	<0.001	-	1.06 [1.04-1.09]	<0.001
Ethnicity						
White	52.7	1.00	-	34.9	1.00	-
Non-white	48.8	1.77 [0.87-3.59]	0.115	61.9	3.79 [1.44-9.94]	0.007
SES						
Higher	53.5	1.00	-	28.9	1.00	-
Intermediate	54.4	1.22 [0.83-1.81]	0.313	37.5	1.78 [1.14-2.79]	0.011
Lower	50.4	1.14 [0.81-1.59]	0.458	38.1	1.73 [1.13-2.66]	0.013
Self-rated health						
Good/very good/excellent	54.3	1.00	-	34.1	1.00	-
Fair/poor	46.4	0.93 [0.65-1.33]	0.688	40.3	1.26 [0.84-1.89]	0.273
BMI	-	0.52 [0.47-0.59]	<0.001	-	0.55 [0.48-0.63]	<0.001
Chronic health conditions						
None	58.8	1.00	-	32.6	1.00	-
≥1	45.8	0.56 [0.41-0.76]	<0.001	37.4	1.08 [0.76-1.55]	0.659

*Indicates the percentage of overweight participants in each group perceiving themselves to be about the right weight or too light.
OR = odds ratio; CI = confidence interval; SES = socioeconomic status.
Weighted data. Sample sizes (*n*) are shown unweighted.

For peer review only

Table 4 Multivariable models testing associations with feeling 'about the right weight' among older men and women with obesity

	Men (*n*=688)			Women (*n*=919)		
	%*	OR [95% CI]	*p*	%*	OR [95% CI]	*p*
Age (years)	-	1.05 [1.01-1.09]	0.013	-	1.04 [1.01-1.08]	0.012
Ethnicity						
White	12.7	1.00	-	8.1	1.00	-
Non-white	5.3	0.32 [0.04-2.48]	0.278	20.7	2.76 [1.03-7.40]	0.043
SES						
Higher	11.1	1.00	-	8.0	1.00	-
Intermediate	11.8	0.96 [0.46-1.98]	0.901	4.5	0.56 [0.23-1.34]	0.190
Lower	13.8	1.34 [0.74-2.43]	0.343	11.0	1.46 [0.74-2.87]	0.272
Self-rated health						
Good/very good/excellent	13.1	1.00	-	8.3	1.00	-
Fair/poor	11.3	0.98 [0.55-1.75]	0.935	9.2	1.01 [0.56-1.83]	0.968
BMI	-	0.76 [0.67-0.87]	<0.001	-	0.96 [0.90-1.03]	0.269
Chronic health conditions						
None	13.9	1.00	-	8.6	1.00	-
≥1	11.6	0.84 [0.48-1.47]	0.541	8.5	0.81 [0.43-1.52]	0.509

*Indicates the percentage of overweight participants in each group perceiving themselves to be about the right weight or too light.

OR = odds ratio; CI = confidence interval; SES = socioeconomic status.

Weighted data. Sample sizes (*n*) are shown unweighted.

For peer review only

Figure legends

Figure 1. The proportion (with 95% confidence interval) of men and women who reported feeling 'too heavy', 'about the right weight', and 'too light', by measured weight status. Weighted data shown.

For peer review only

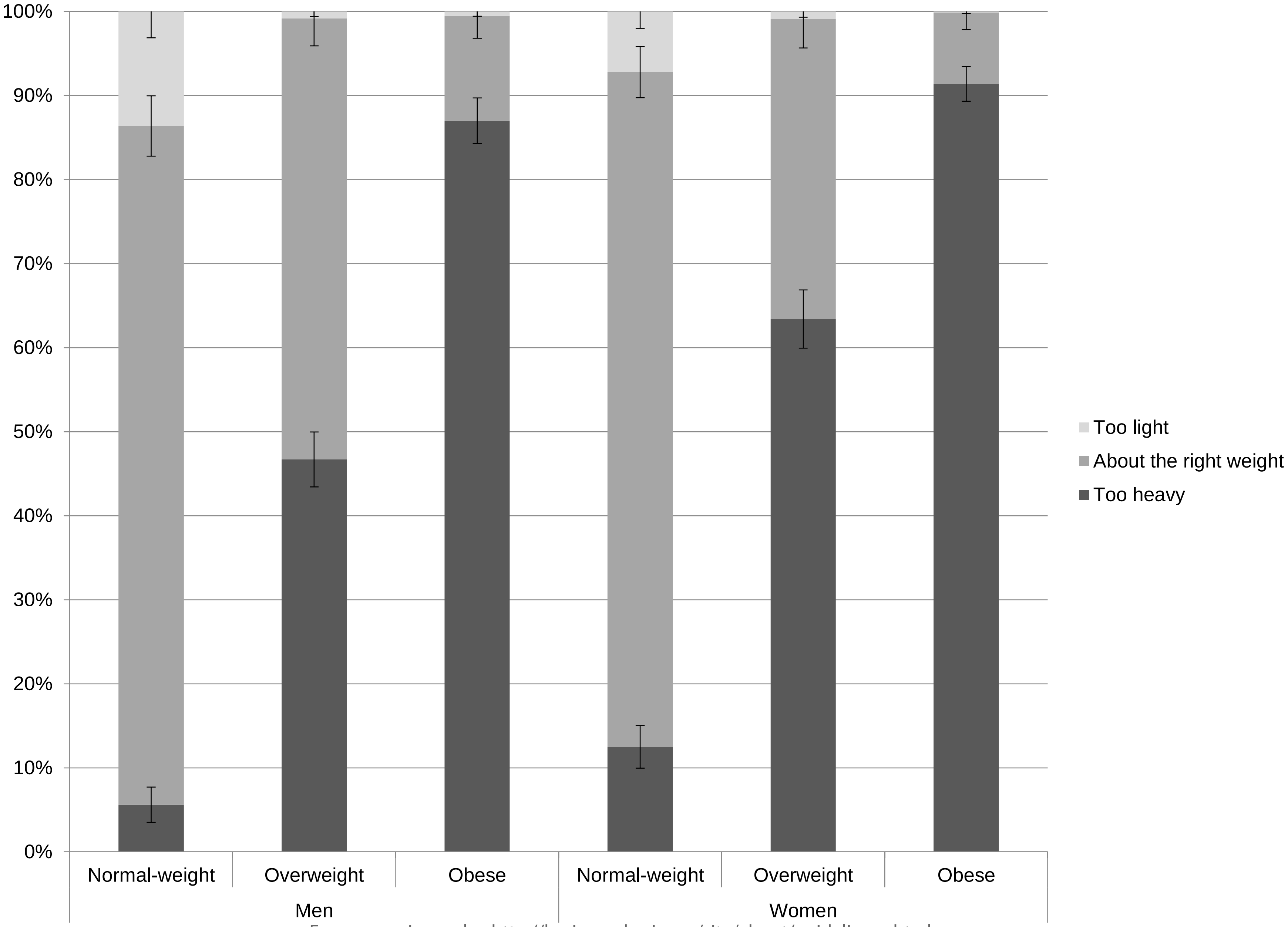
100%
90%
80%
70%
60%
50%
40%
30%
20%
10%
0%
Normal-weight
Overweight
Obese
Normal-weight
Overweight
Obese
Men
Women
Too light
About the right weight
Too heavy

STROBE Statement—Checklist of items that should be included in reports of ***cross-sectional studies***

	Item No	Recommendation	Page No
**Title and abstract**	1	(*a*) Indicate the study's design with a commonly used term in the title or the abstract	2
		(*b*) Provide in the abstract an informative and balanced summary of what was done and what was found	2
**Introduction**			
Background/rationale	2	Explain the scientific background and rationale for the investigation being reported	4-5
Objectives	3	State specific objectives, including any prespecified hypotheses	5
**Methods**			
Study design	4	Present key elements of study design early in the paper	5
Setting	5	Describe the setting, locations, and relevant dates, including periods of recruitment, exposure, follow-up, and data collection	5
Participants	6	(*a*) Give the eligibility criteria, and the sources and methods of selection of participants	5
Variables	7	Clearly define all outcomes, exposures, predictors, potential confounders, and effect modifiers. Give diagnostic criteria, if applicable	5-6
Data sources/ measurement	8*	For each variable of interest, give sources of data and details of methods of assessment (measurement). Describe comparability of assessment methods if there is more than one group	*5-6*
Bias	9	Describe any efforts to address potential sources of bias	6-7
Study size	10	Explain how the study size was arrived at	5
Quantitative variables	11	Explain how quantitative variables were handled in the analyses. If applicable, describe which groupings were chosen and why	5-6
Statistical methods	12	(*a*) Describe all statistical methods, including those used to control for confounding	6-7
		(*b*) Describe any methods used to examine subgroups and interactions	6-7
		(*c*) Explain how missing data were addressed	6-7
		(*d*) If applicable, describe analytical methods taking account of sampling strategy	6
		(*e*) Describe any sensitivity analyses	na
**Results**			
Participants	13*	(a) Report numbers of individuals at each stage of study—eg numbers potentially eligible, examined for eligibility, confirmed eligible, included in the study, completing follow-up, and analysed	5
		(b) Give reasons for non-participation at each stage	Na
		(c) Consider use of a flow diagram	Na
Descriptive data	14*	(a) Give characteristics of study participants (eg demographic, clinical, social) and information on exposures and potential confounders	7
		(b) Indicate number of participants with missing data for each variable of interest	Tables
Outcome data	15*	Report numbers of outcome events or summary measures	Tables
Main results	16	(*a*) Give unadjusted estimates and, if applicable, confounder-adjusted estimates and their precision (eg, 95% confidence interval). Make clear which confounders were adjusted for and why they were included	Tables

For peer review only - http://bmjopen.bmj.com/site/about/guidelines.xhtml

		(*b*) Report category boundaries when continuous variables were categorized	Tables
		(*c*) If relevant, consider translating estimates of relative risk into absolute risk for a meaningful time period	Na
Other analyses	17	Report other analyses done—eg analyses of subgroups and interactions, and sensitivity analyses	na
**Discussion**			
Key results	18	Summarise key results with reference to study objectives	9-10
Limitations	19	Discuss limitations of the study, taking into account sources of potential bias or imprecision. Discuss both direction and magnitude of any potential bias	11
Interpretation	20	Give a cautious overall interpretation of results considering objectives, limitations, multiplicity of analyses, results from similar studies, and other relevant evidence	11-12
Generalisability	21	Discuss the generalisability (external validity) of the study results	11
**Other information**			
Funding	22	Give the source of funding and the role of the funders for the present study and, if applicable, for the original study on which the present article is based	17

*Give information separately for exposed and unexposed groups.

Note: An Explanation and Elaboration article discusses each checklist item and gives methodological background and published examples of transparent reporting. The STROBE checklist is best used in conjunction with this article (freely available on the Web sites of PLoS Medicine at http://www.plosmedicine.org/, Annals of Internal Medicine at http://www.annals.org/, and Epidemiology at http://www.epidem.com/). Information on the STROBE Initiative is available at www.strobe-statement.org.

For peer review only
